# Colonial drivers and cultural protectors of brain health among Indigenous peoples internationally

**DOI:** 10.3389/fpubh.2024.1346753

**Published:** 2024-02-07

**Authors:** Rita Henderson, Joyla A. Furlano, Shayla Scott Claringbold, Ashley Cornect-Benoit, Anh Ly, Jennifer Walker, Lisa Zaretsky, Pamela Roach

**Affiliations:** ^1^Department of Family Medicine, University of Calgary, Calgary, AB, Canada; ^2^Schulich School of Medicine and Dentistry, University of Western Ontario, London, ON, Canada; ^3^Department of Community Health Sciences, University of Calgary, Calgary, AB, Canada; ^4^Department of Psychology, University of Calgary, Calgary, AB, Canada; ^5^Department of Health Research Methods, Evidence and Impact, McMaster University, Hamilton, ON, Canada

**Keywords:** Indigenous, brain health, cognition, dementia, Alzheimer’s disease, social, culture, scoping review

## Abstract

Despite relatively higher rates of dementia among Indigenous populations internationally, research into drivers of disparities in brain health and cognitive function has tended to focus on modifiable risk factors over cultural understandings and contextual determinants. By seeking to characterize social and cultural factors that shape brain health and cognition in Indigenous populations, this mini scoping review expands prevailing schools of thought to include Indigenous knowledge systems. This reveals important gaps in culturally aligned care. It also reclaims horizons for research important to Indigenous Peoples that have garnered diminished attention in biomedical approaches. Twenty-three sources were included for data extraction. This synthesis of 23 sources includes health communication about dementia, health provider knowledge about Indigenous health, culturally relevant screening and assessment tools, and culturally grounded care models. Much of the focus is currently still on modifiable risk factors that reside at individual factors, whereas attention to wider social factors that impact populations is needed, as stressors through isolation, discrimination, and unequal care are widely reported. Going forward, identifying structural barriers to living well and recognizing the importance of connection to culture will benefit both Indigenous and non-Indigenous understandings of brain health.

## 1 Introduction

Colonization and ongoing colonial systems and structures continue to impact the health and wellness of Indigenous Peoples. Growing evidence indicates that Indigenous people experience higher risk of cognitive impairment, including dementia. Walker and colleagues (1) note how, in the international context, rates of Alzheimer’s disease and related dementias (ADRDs) appear to be rising more rapidly in Indigenous compared to non-Indigenous populations. This disparity is concerning given that the proportion of people with an ADRD diagnosis across all populations is projected to more than double over the next 25 years (2, 3), meaning a greater proportion of Indigenous Peoples of all ages may develop cognitive impairment. For instance, the prevalence of dementia in First Nations in the Canadian province of Alberta in 2009 was 7.5 per 1,000 compared to non-First Nations people at 5.6 per 1,000 (4). Moreover, evidence suggests that onset of dementia occurs at a younger age and that rates are rising more quickly for First Nations individuals, disproportionately affecting males (4).

While epidemiological research sheds light on prevalence and incidence rates of dementia among Indigenous populations ranging between 0.5 to 20% (5), explanatory frameworks for these are not fully developed. Little is known about social factors that may affect brain health and cognition that are specific to Indigenous communities with shared experiences of colonial oppression (6). Determinants of health known to play an important role in shaping Indigenous Peoples’ experiences of health more generally include socioeconomic status; history of colonization; systemic racism; cultural perspectives and practices; healthcare access; and importantly, safety. Indigenous cultural perspectives on cognitive impairment and the aging process have garnered increasing attention (7–9), while other researchers focus on culturally grounded caregiving models (10) and cross-cultural communication and patient health education (11). While growing attention is on social and cultural factors that influence Indigenous Peoples’ experiences of cognitive health, more research is needed. In one recent integrative review of literature on Indigenous perspectives on cognitive impairment and dementia, Racine and colleagues (12) concluded that a dearth of published information exists about Indigenous perspectives on aging or experiences of cognitive impairment and dementia. Racine et al. (12) urged researchers to more carefully examine the role of Indigenous knowledge, culture, and traditions in this domain, in order to decolonize dementia care.

We are a team of Indigenous and allied scholars and research associates located across what is now known as Canada, all affiliated with the Canadian Consortium on Neurodegeneration in Aging (CCNA). With several Indigenous co-authors, we address an erasure bias in Western scientific approaches that often minimize consideration of social and cultural factors to health and disease outcomes. The wider Indigenous cognitive health research team in the CCNA is organized around priority focus areas of advancing knowledge around the appropriate diagnosis of brain-related conditions in Indigenous populations, improved care for Indigenous Peoples with such conditions, and better understanding social and cultural realities that interface with brain health and cognitive function. Our work is generally community-based, and from this location this scoping review was an opportunity to bring together our collective learnings and knowledge. The objective of our scoping review is to characterize social and cultural factors that affect brain health and cognition with Indigenous populations, with particular interest in community understandings of these.

## 2 Methods

Scoping reviews are appropriately and commonly used to identify gaps in knowledge or explore the nature of published knowledge on a topic (13). Following Arksey & O’Malley’s (14) six-part scoping review methodology and the PRISMA reporting standards for scoping reviews, we conducted a search of CINAHL Plus, Ovid Medline, EMBASE, SocINDEX, Scopus, and the Canadian Research Index from January 26 to February 1, 2022.

### 2.1 Search strategy

The search strategy was designed to retrieve social and cultural drivers of brain health and cognition among Indigenous Peoples. The team consulted with health librarians and experts in brain health and Indigenous health to supplement our own expertise in these areas. Recalling that the main shared experience of Indigenous Peoples internationally is colonization, our search focused on dual aspects of social worlds shaped by settler colonial systems: Indigenous cultures and their disruption by colonization. On one hand, this includes cultural practices, perspectives, and knowledge around the brain and its health, and on the other hand social environments characterized by oppression such as stigma, racism, marginalization, and resource disparities. Key search terms included those related to these social factors, brain and cognitive health, and Indigenous identities. No geographic restriction was imposed, nor a limit on the year of publication. Supplementary Table 1 displays key search terms.

### 2.2 Source selection

Sources needed to meet the following criteria for inclusion: (1) include an Indigenous population (‘Indigenous’ as defined by the United Nations Declaration on the Rights of Indigenous Peoples) (15); (2) include findings focused on brain health or cognition (including dementia or other neurodegenerative disorders); (3) discuss social or cultural factors (e.g., discrimination, socioeconomic disparities, social mobility, language) that may drive brain or cognitive health outcomes; and (4) be written in English. Two reviewers (SSC and JAF) independently conducted an initial screen of studies exported from the searches to the review management platform COVIDENCE, in the first stage reviewing only titles and abstracts for eligible inclusion. This was followed by a full-text scan of documents for eligibility conducted by the same reviewers. Sources were excluded if they were: (1) textbooks or handbooks (i.e., only providing a summary of existing work); (2) systematic and scoping review papers (i.e., not reporting original source material); (3) abstracts only; (4) animal research; (5) not Indigenous-focused (i.e., Indigenous Peoples appearing only as a comparator group among several); (6) no reference to social or cultural factors driving factors of brain health or cognition; or (7) outcomes were mental health disorders as defined by the Diagnostic and Statistical Manual of Mental Disorders (DSM-V-TR) due to our focus on brain health or cognition, not mental health. At this stage to identify further relevance, we also accessed titles and abstracts of sources that may have been missed in the original searches, reference lists from included studies (reviewed by SSC), with potentially relevant sources also assessed for title and abstract. A full-text review was then conducted by both reviewers (SSC and JAF). Individual results were compared and consensus of final inclusion of sources reached in discussion with the full project team.

### 2.3 Data extraction and analysis

Two reviewers (SSC and JAF) used a common data extraction form in Excel. Data fields included, when applicable: author(s), year of publication, article type, design, study date, sample, population demographics (age, sex/gender), details on Indigenous population included, geographic location, project collaborations (e.g., with community organizations), study objectives, social or cultural factors discussed, brain health or cognition outcome(s) examined, presentation of research rigor, findings, recommendations, and reported limitations. Special attention was paid to sex- or gender-reported data during extraction, reporting where relevant. Additionally, we searched each paper using the terms ‘sex’, ‘gender’, ‘male’, ‘female’, ‘men’, ‘women’, ‘man’, and ‘woman’ to ensure that no pertinent data was overlooked. This was undertaken to anticipate meaningful sex- or gender-based observations in or across included studies.

The wider author team then conducted a series of three consensus-building discussions based on a Nominal Group Technique (NGT), a structured small-group discussion method to reach consensus through brainstorming to identify and interpret emergent themes (16). Bringing together perspectives from our CCNA Indigenous cognitive health team, we generated an exhaustive list of observed categories in the data, then discussed what resonated and what was surprising in order to structure results presented here. Lead authors (RH and PR) then worked with transcripts from NGT discussions to discuss findings according to the Knowledge-Attitudes-Behavior (KAB) model (adapted from Knowledge-Attitudes-Practices [KAP] Model, where “practices” may be more narrowly medical than “behaviours”). Research suggests that the KAB model is often helpful for health behavior change theory (17). This offers a framework for organizing behavior change theory for health audiences, helping to orient the implications of findings for our colleagues in clinical practice, planning, and education. We take some liberty with the KAB model, as the inter-cultural nature of data presented here means that we treat “knowledge” within a constructivist lens (18), and “attitudes” may also reflect values or cultural principles.

## 3 Results

The search of the combined databases yielded 661 initial results. Sources were imported to COVIDENCE, where 131 were identified as duplicates and removed. From there, 530 underwent abstract and title screening, where 444 were excluded (Supplementary Figure 1). Eighty-six articles underwent full-text review, leading to an additional 72 being further excluded. Ultimately, 15 sources were identified as relevant from the initial database search. An additional 14 sources were identified as possibly relevant through hand-searching reference lists of eligible sources. These additional 14 underwent title, abstract and full-text screening, where eight were identified as eligible. A total of 23 articles were included for data extraction. Of these, the majority are in Australia (*n* = 8), followed by United States (*n* = 5), Canada (*n* = 4), New Zealand (*n* = 3), Guam (*n* = 2), and Malayasia (*n* = 1). The majority of articles included in the scoping review are quantitative in nature (*n* = 8), followed by commentaries (*n* = 7), qualitative methods (*n* = 5), and mixed methods (*n* = 3). Supplementary Figure 1 displays the PRISMA flow diagram.

Given the cultural and social diversity of Indigenous groups internationally, comparative differences are not suitable to link to explanatory frameworks here, though commonalities of primary interest. For the purpose of reporting findings, identified themes are broadly divided into categories of social versus cultural drivers impacting brain health and cognition. Key areas examined in this review include health communication about dementia, health provider knowledge about Indigenous health, culturally relevant dementia-screening and assessment tools, and culturally grounded care models. Important to note is a lack of sex and gender considerations found in the literature, suggesting more research is required (19).

### 3.1 Social drivers impacting brain health and cognition for Indigenous peoples

Fifteen articles identified social factors that affect brain health or cognition with Indigenous populations (10, 20–33). Studies acknowledged that population health research has historically focused on modifiable risk factors which include smoking, physical inactivity, and low educational attainment associated with dementia (24, 26, 30). Some note that the literature has begun to describe complexities in modifiable risk factors, such as education and occupation (21, 25, 27, 28). For instance, Radford et al. (28) compared skilled with unskilled employment (e.g., labor or entry-level jobs), identifying that many patients diagnosed with dementia had an occupational history of unskilled work. Three articles addressed low income as a risk factor for brain or cognitive health outcomes; however, the relationship was not explored as extensively as educational attainment (24, 26, 30). The inter-related connections between educational attainment, employment and other modifiable risk factors for dementia remains largely unexplored, especially within the context of Indigenous Peoples’ health.

Several studies identified the impacts of colonization (10), institutional racism (31) and historical and ongoing trauma (9) as drivers of brain health or cognition. Radford et al. (28) measured cultural experiences and informal education using a scale entitled Retrospective Indigenous Childhood Enrichment (RICE). The RICE tool was developed to approximate cognitive stimulation throughout childhood outside of school in the Aboriginal Australian population. The paper also addresses childhood trauma as measured by the childhood trauma questionnaire (CTQ), a survey quantifying adverse childhood experiences ranging from separation from family to socioeconomic status. The CTQ survey indicated an association between childhood stress and late-life dementia diagnosis in Aboriginal Australian populations (28). Additional work to identify links between early childhood experiences and cognitive decline and dementia in adulthood are necessary to further understand the relationship between these outcomes.

Social impact and climate change were presented as a factor influencing Indigenous brain health in many papers. Data from Guam measured cycad exposure during traditional food gathering, preparation, and consumption of an Indigenous food called fadang, exploring this as a risk factor for neurodegenerative diseases (34). Although the association between fadang consumption was inconclusive, the study probes population-specific lifestyle factors such as exposure to eating fadang in young adulthood, highlighting potential sex differences to be further explored. Southern Inuit research participants from Eastern Canada identified dementia prevention as a small piece in the broader category of healthy aging (9). Participants in this study advocated for more access to foods associated with traditional diets in the region, such as wild game and locally-grown berries. Disruption in the food chain due to environmental contamination and colonization has interrupted access to traditional foods, simultaneously interrupting opportunities to engage with the land – which was considered in the study community to be protective of mental wellness (9). A commentary by Farugia et al. (22) linked access to traditional food and Indigenous brain health to extreme heat. The authors explored the link between bushfires, access to care in rural environments, and the exacerbation of neurodegeneration among dementia patients exposed to extreme heat. Similarly, other work has highlighted that urbanization and climate change, including issues of urban migration, family structure, and community involvement, can have a lasting impact on brain health and dementia (10).

### 3.2 Cultural drivers impacting brain health and cognition for indigenous peoples

Eleven articles identified cultural factors that affect brain health or cognition in Indigenous populations (9, 10, 22, 25, 34–40). Some sources address physiological and functional symptoms resulting from the differences and tensions that lie between Indigenous perspectives and biomedical approaches (9, 37, 39). Pace (9) identified the increasing difficulty of being able to live in traditional ways on the land in one’s own territory. This was seen to create inequity in who may receive needed services and thereby be able to age “in place,” which the study authors note is often recommended as best practice. Additionally, findings indicate that place and culture foster identity maintenance, and are protective factors against cognitive decline (9). Similarly, Manly and Espino (39) outlined how culture shapes cognitive function and affects potential presentation of brain aging clinically, impacting efforts to diagnose and manage cognitive decline across groups. Both studies disrupt conventional western and biomedical parameters defining neurodegenerative conditions of the brain by highlighting protective aspects of cultural connection. This highlights the need for flexible and personalized diagnostic approaches inclusive of linguistic and epistemological differences across ethno-cultural groups (38).

Cultural understandings of dementia were noted to shape expectations of healthcare and influence preferences for informal caregiving in community networks over accessing formal caregiving services (10). Some sources outlined efforts to embed cultural norms within Western healthcare systems, while others emphasized that not all Indigenous Peoples may desire to access Westernized care as their conditions progress (22, 25, 35, 38). These studies note reasons anchored in cultural perceptions of the purpose of care itself for brain aging. One study illustrated this by contrasting Hawai’ian expectations that care focus on personalized support defined by an individual and their family or community, where a Western healthcare approach tends to emphasize mitigating all disease symptoms in the individual (35).

Most studies used sex and gender interchangeably and did not use these variables in a meaningful way. On study looked at differences in exposure to cycad and dementia risk by sex (34). Few papers (20, 28–30) looked at risk or predictive factors of dementia or cognitive decline based on sex or gender. This indicates a need for future research to intentionally include sex and gender analyses when undertaking primary research with Indigenous people living with dementia.

## 4 Discussion

Our review has highlighted the presence of structural drivers, both social and cultural, of Indigenous brain health and cognition. Much discourse in current evidence is shifting to focus on modifiable risk factors (i.e., childhood stressors). This emphasis nevertheless tends to neglect the influence of colonial structures on the autonomy of Indigenous Peoples to control these modifiable risk factors, such as income status or ability to remain in home territory or community. Instead, community-aligned perspectives reviewed here suggest critical attention be directed at systemic, structural, and social determinants of health, rather than individual ones. Large-scale policy and legislative changes to enshrine self-governance and self-determination in health and social care systems will be an important future change to create these solutions. Stites et al. (41) have established a framework for gathering structural and social determinants of health in ADRDs research which recognizes the importance of cultural values and perspectives. This may offer a crucial opportunity to identify structural barriers in order to decolonize and dismantle them.

The path to decolonizing brain health supports and care for Indigenous Peoples, therefore, includes emphasis on strengths of connection to culture. This includes cultural perspectives on brain aging encountered by many on our research team, who expected from our own community-based experiences to find in this review Indigenous community hesitation to defining brain aging simply in terms of disease and decline in favor of treating it as a natural component of the life course, where those exhibiting cognitive challenges may retain place and purpose in their social worlds. Embedding understanding of culture and social context will benefit all populations, Indigenous and non-Indigenous. For example, the Educating for Equity Care Framework developed for physicians providing care to Indigenous Peoples with diabetes, highlights colonization as a major health inequity and poses that while respecting diverse perspectives and experiences, culture “is a facilitator of the clinical relationship and patient capacity” (42, 43). Distinctions also need to be considered to avoid pan-Indigenizing populations. Understanding how cultural understandings of dementia and aging can differ among communities is imperative when developing appropriate responses. This approach is in alignment with the intention of Shkaabe Makwa (44), a team focused on driving culturally relevant system initiatives to achieve health equity and community wellness among First Nations, Métis, and Inuit populations. In addition, given that brain aging and cognition share many determinants with mental health outcomes, such as life stressors as risk factors and social connectedness as protective, the First Nations Mental Wellness Continuum Framework provides guidance and direction for how to appropriately embed cultural, social, and system factors (45).

In summary, listening to the experiences and asks of community members takes time, compassion, and meaningful relationship development. Sources reviewed here emphasize that it is imperative that dementia care be culturally safe and designed in collaboration with Indigenous Peoples themselves (19). Future research would do well to acknowledge that health and wellness extend beyond the biomedical model. This will aid in facilitating pathways that wholistically address the impact of bio-psycho-socio-cultural factors, though gaps in bridging evidence between these domains for improved care approaches clearly persist. This requires a willingness to learn and critically understand Indigenous values and principles broadly and locally, knowing that Indigenous-centered brain healthcare cannot be essentialized or reduced into universalized models of care. Studies discussed here highlight the importance of culture, family, community, respect, and trauma-informed care in ensuring that Indigenous Peoples’ brain health and cognition are appropriately supported (46). Finally, the Truth and Reconciliation Commission of Canada: Calls to Action and the United Nations Declaration on the Rights of Indigenous Peoples (UNDRIP) are guides that draw attention to the inherent right for self-determination in healthcare, and as such are tools for affirming person, family, and community-centered.

## Author contributions

RH: Writing – original draft, Writing – review & editing. JF: Writing – original draft, Writing – review & editing. SC: Writing – original draft, Writing – review & editing. AC-B: Writing – original draft, Writing – review & editing. AL: Writing – original draft, Writing – review & editing. JW: Writing – original draft, Writing – review & editing. LZ: Writing – original draft, Writing – review & editing PR: Writing – original draft, Writing – review & editing.

## References

[1] Walker J Spiro G Loewen K Jacklin K. Alzheimer's disease and related dementia in indigenous populations: a systematic review of risk factors. J Alzheimer's disease: JAD. (2020) 78:1439–51. doi: 10.3233/JAD-200704, PMID: 33185601

[2] Alzheimer Society of Canada. Race and dementia. (2020). Available at: https://alzheimer.ca/en/take-action/change-minds/race-dementia [Accessed September 1, 2023].

[3] Public Health Agency of Canada. A dementia strategy for Canada: together we aspire. (2019). Available at: https://www.canada.ca/en/public-health/services/publications/diseases-conditions/dementia-strategy.html [Accessed September 3, 2023].

[4] Jacklin KM Walker JD Shawande M. The emergence of dementia as a health concern among first nations populations in Alberta. Canada Canadian J Public Heal. (2012) 104:e39–44. doi: 10.1007/BF03405652, PMID: 23618107 PMC6973818

[5] Warren LA Shi Q Young K Borenstein A Martiniuk A. Prevalence and incidence of dementia among indigenous populations: a systematic review. Int Psychogeriatr. (2015) 27:1959–70. doi: 10.1017/S1041610215000861, PMID: 26088474

[6] King M Smith A Gracey M. Indigenous health part 2: the underlying causes of the health gap. Lancet (British Edition). (2009) 374:76–85. doi: 10.1016/S0140-6736(09)60827-8, PMID: 19577696

[7] Hulko W Camille E Antifeau E Arnouse M Bachynski N Taylor D. Views of first nation elders on memory loss and memory care in later life. J Cross Cult Gerontol. (2010) 25:317–42. doi: 10.1007/s10823-010-9123-9, PMID: 20593232

[8] Johnston K Preston R Strivens E Qaloewai S Larkins S. Understandings of dementia in low- and middle-income countries and amongst indigenous peoples: a systematic review and qualitative meta-synthesis. Aging Ment Health. (2020) 24:1183–95. doi: 10.1080/13607863.2019.1606891, PMID: 31074290

[9] Pace J. “Place-ing” dementia prevention and care in NunatuKavut. Labrador Canadian J Aging. (2020) 39:247–62. doi: 10.1017/S0714980819000576, PMID: 31666149

[10] Jacklin K Pace JE Warry W. Informal dementia caregiving among indigenous communities in Ontario. Canada Care Manag J. (2015) 16:106–20. doi: 10.1891/1521-0987.16.2.106, PMID: 26171510

[11] Webkamigad S Rowe R Peltier S Froehlich CA McGilton KS. Identifying and understanding the health and social care needs of indigenous older adults with multiple chronic conditions and their caregivers: a scoping review. BMC Geriatr. (2020) 20:145–5. doi: 10.1186/s12877-020-01552-5, PMID: 32306912 PMC7168986

[12] Racine L Johnson L Fowler-Kerry S. An integrative review of empirical literature on indigenous cognitive impairment and dementia. J Adv Nurs. (2021) 77:1155–71. doi: 10.1111/jan.14665 33270270

[13] Munn Z Peters MDJ Stern C Tuganaru C McArthur A Aromataris E. Systematic review or scoping review? Guidance for authors when choosing between a systematic or scoping review approach. BMC Med Res Methodol. (2018) 18:143. doi: 10.1186/s12874-018-0611-x 30453902 PMC6245623

[14] Arksey H O'Malley L. Scoping studies: towards a methodological framework. Int J Soc Res Method. (2005) 8:19–32. doi: 10.1080/1364557032000119616

[15] United Nations Declaration on the Rights of Indigenous Peoples. (2008). Available at: http://www.un.org/esa/socdev/unpfii/documents/DRIPS_en.pdf. [Accessed September 3, 2023].

[16] Centers for Disease Control and Prevention. Gaining consensus among stakeholders through nominal group technique. (2018). Available at: https://www.cdc.gov/healthyyouth/evaluation/pdf/brief7.pdf. [Accessed May 15, 2023].

[17] Schrader PG Lawless KA. The knowledge, attitudes, & behaviors approach how to evaluate performance and learning in complex environments. Perform Improvt (Int Society for Perform Improv). (2004) 43:8–15. doi: 10.1002/pfi.4140430905

[18] Charmaz K. The power of constructivist grounded theory for critical inquiry. Qual Inq. (2017) 23:34–45. doi: 10.1177/1077800416657105

[19] Chakanyuka C Bacsu JDR DesRoches A Dame J Carrier L Symenuk P. Indigenous-specific cultural safety within health and dementia care: a scoping review of reviews. Soc Sci Med. (2022) 293:114658. doi: 10.1016/j.socscimed.2021.114658 34942579

[20] de Kok DS Teh RO Pillai A Connolly MJ Wilkinson TJ Jacobs R. What is the relationship between visual impairment and cognitive function in octogenarians? N Z Med J. (2017) 130:33–47. PMID: 28796770

[21] Dyall L. Dementia: continuation of health and ethnic inequalities in New Zealand. N Z Med J. (2014) 127:68–80. PMID: 24548958

[22] Farugia TL Cuni-Lopez C White AR. Potential impacts of extreme heat and bushfires on dementia. J Alzheimer’s disease: JAD. (2021) 79:969–78. doi: 10.3233/JAD-201388, PMID: 33459654

[23] Garrett MD Baldridge D Benson W Crowder J Aldrich N. Mental health disorders among an invisible minority: depression and dementia among American Indian and Alaska native elders. Gerontologist. (2015) 55:227–36. doi: 10.1093/geront/gnu181, PMID: 26035598

[24] MacDonald JP Barnes DE Middleton LE. Implications of risk factors for Alzheimer’s disease in Canada’s indigenous population. Canadian Geriatrics J. (2015) 18:152–8. doi: 10.5770/cgj.18.159, PMID: 26495049 PMC4597815

[25] Pollitt PA. The problem of dementia in Australian aboriginal and Torres Strait islander communities: an overview. Int J Geriatr Psychiatry. (1997) 12:155–63. doi: 10.1002/(SICI)1099-1166(199702)12:2<155::AID-GPS582>3.0.CO;2-M, PMID: 9097208

[26] Puun BI Othman Z Drahman I. Dementia among elderly Melanau: a community survey of an indigenous people in East Malaysia. Intern Med J. (2014) 21:468–71.

[27] Radford K Delbaere K Draper B Mack HA Daylight G Cumming R. Childhood stress and adversity is associated with late-life dementia in aboriginal Australians. Am J Geriatr Psychiatry. (2017) 25:1097–106. doi: 10.1016/j.jagp.2017.05.008, PMID: 28689644

[28] Radford K Lavrencic LM Delbaere K Draper B Cumming R Daylight G. Factors associated with the high prevalence of dementia in older aboriginal Australians. J Alzheimers Dis. (2019) 70:S75–85. doi: 10.3233/JAD-180573, PMID: 30507573 PMC6700619

[29] Reed DM Brody JA. Amyotrophic lateral sclerosis and parkinsonism dementia on Guam, 1945-1972. 1. Descriptive epidemiology. Am J Epidemiol. (1975) 101:287–301. doi: 10.1093/oxfordjournals.aje.a112097, PMID: 1124759

[30] Smith K Flicker L Dwyer A Atkinson D Almeida OP Lautenschlager NT. Factors associated with dementia in aboriginal Australians. Aust N Z J Psychiatry. (2010) 44:888–93. doi: 10.3109/00048674.2010.491816, PMID: 20932202

[31] Shepherd SM Ogloff JRP Shea D Pfeifer JE Paradies Y. Aboriginal prisoners and cognitive impairment: the impact of dual disadvantage on social and emotional wellbeing. J Intellect Disabil Res. (2017) 61:385–97. doi: 10.1111/jir.12357, PMID: 28054417

[32] Glasson EJ Sullivan SG Hussain R Bittles AH. An assessment of intellectual disability among aboriginal Australians. J Intellect Disabil Res. (2005) 49:626–34. doi: 10.1111/j.1365-2788.2005.00722.x, PMID: 16011555

[33] Lo Giudice D Smith K Fenner S Hyde Z Atkinson D Skeaf L. Incidence and predictors of cognitive impairment and dementia in aboriginal Australians: a follow-up study of 5 years. Alzheimers Dement. (2016) 12:252–61. doi: 10.1016/j.jalz.2015.01.009, PMID: 25998515

[34] Borenstein AR Mortimer JA Schofield E Wu Y Salmon DP Gamst A. Cycad exposure and risk of dementia, MCI, and PDC in the Chamorro population of Guam. Neurology. (2007) 68:1764–71. doi: 10.1212/01.wnl.0000262027.31623.b2, PMID: 17515538

[35] Braun KL Browne CV. Perceptions of dementia, caregiving, and help seeking among Asian and Pacific islander American. Health Soc Work. (1998) 23:262–74. doi: 10.1093/hsw/23.4.262, PMID: 9834879

[36] Cullum S Varghese C Coomarasamy C Whittington R Hadfield L Rajay A. Predictors of mortality in Māori, Pacific Island, and European patients diagnosed with dementia at a New Zealand memory service. Int J Geriatr Psychiatry. (2020) 35:516–24. doi: 10.1002/gps.5266, PMID: 31957058

[37] Laditka JN Laditka SB Liu R Price AE Wu B Friedman DB. Older adults’ concerns about cognitive health: commonalities and differences among six United States ethnic groups. Ageing & Society. (2011) 31:1202–28. doi: 10.1017/S0144686X10001273

[38] Laditka SB Laditka JN Liu R Price AE Friedman DB Wu B. How do older people describe others with cognitive impairment? A multiethnic study in the United States. Ageing & Society. (2013) 33:369–92. doi: 10.1017/S0144686X11001255

[39] Manly JJ Espino DV. Cultural influences on dementia recognition and management. Clin Geriatr Med. (2004) 20:93–119. doi: 10.1016/j.cger.2003.10.004, PMID: 15062490

[40] Seto J Davis J Taira DA. Examining the association between different aspects of socioeconomic status, race, and disability in Hawaii. J Racial Ethn Health Disparities. (2018) 5:1247–53. doi: 10.1007/s40615-018-0471-4, PMID: 29464658

[41] Stites SD Midgett S Mechanic-Hamilton D Zuelsdorff M Glover CM Marquez DX. Establishing a framework for gathering structural and social determinants of health in Alzheimer’s disease research centers. Gerontologist. (2022) 62:694–703. doi: 10.1093/geront/gnab182, PMID: 34919705 PMC9154263

[42] Crowshoe LL Henderson R Jacklin K Calam B Walker L Green ME. Educating for equity care framework: addressing social barriers of indigenous patients with type 2 diabetes. Can Fam Physician. (2019) 65:25–33. PMID: 30674510 PMC6347314

[43] World Alzheimer Report. Chapter 20. Sex, gender and cultural factors. (2021). Available at: https://www.alzint.org/u/World-Alzheimer-Report-2021-Chapter-20.pdf [Accessed November 6, 2023)].

[44] CAMH. Shkaabe-makwa. (2023). Available at: https://www.camh.ca/en/driving-change/shkaabe-makwa. [Accessed September 25, 2023].

[45] Thunderbird Partnership Foundation. First nations mental wellness continuum framework. (2022). Available at: https://thunderbirdpf.org/fnmwc/ [Accessed November 1, 2023].

[46] LoGiudice D. Hughson J. Douglas H. Wenitong M. Belfrage M. Culturally safe, trauma informed approach to cognitive impairment and dementia in older aboriginal and Torres Strait islander people [review of culturally safe, trauma informed approach to cognitive impairment and dementia in older aboriginal and Torres Strait islander people]. (2022). 505–511. New York: New York Times Company. 10.31128/AJGP-01-23-6672 37532438

