# Supplementary material for: Colonial drivers and cultural protectors of brain health among Indigenous peoples internationally
Source: Front Public Health. 2024 Feb 7;12:1346753. doi: 10.3389/fpubh.2024.1346753 (PMC10903363; doi:10.3389/fpubh.2024.1346753)
Supplement: Supplementary file 1 [file Table_1.DOCX]

Table 1 Key Search Terms

| Social | Cognition | Indigen* |
| --- | --- | --- |
| Stigma | “Brain structure” | Aboriginal |
| Racism | “Brain function” | “First Nations” |
| Discrimination | “Brain health” | Northern |
| Colonization | “Cognition disorder” | Native |
| Marginalization | “Brain injury” | Inuit |
| Socioeconomic | “Neurocognitive disorder” | Métis |
| Policy | “Cognitive impairment” | Amerindian |
| Unemployment | Dementia | “Torres strait” |
| “Social isolation” |  | Hawaiian |
| “Social determinant* health” |  | Pacific Islander |
| “Social mobility”/class |  | Maori |
| “Social conditions” |  | Circumpolar |
| Cultural |  |  |


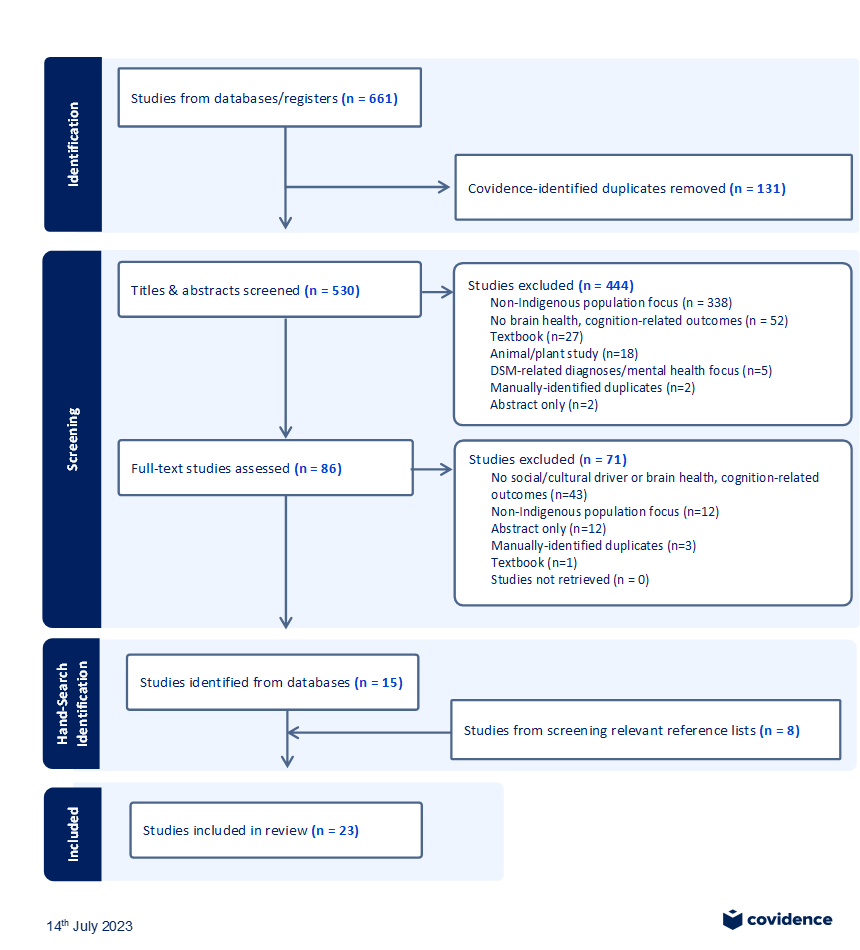


**Supplementary Figure 1.** PRIMSA Flow Diagram.
